# New Appliances and Things Medical

**Published:** 1902-10-25

**Authors:** 


### NEW APPLIANCES AND THINGS MEDICAL.

**[We shall be glad to receive at our Office, 28 & 29 Southampton Street, Strand, London, W.C., from the manufacturers, specimens of all new preparations and appliances which may be brought out from time to time.]**

### MANHU DIABETIC FOODS.

(The Manhu Food Company, Ltd., Vauxhill Mills, Liverpool, and 88 Charing Cross Road, London, W.C.)

The Manhu diabetic foods consist of flour, biscuits, flaked wheat, flaked barley, prepared barley, barley cocoa, and macaroni. All these substances consist of cereals specially treated and, it is claimed, so molecularly altered as to become capable of being assimilated by a diabetic patient and utilised by the system—that is to say, it is assumed that the carbohydrates of which the Manhu preparations consist are not excreted by the kidneys in the form of sugar, but that they are of economic value, and oxidised, as is the case with healthy individuals, into carbonic acid and water. Without entering into the controversial aspects of this claim urged by the proprietors of this food, we may state at once that the Manhu preparations are easily digested and assimilated, and that we believe a diabetic patient would, in the long run, more reasonably consider his health and prospects of life by a strictly limited diet of Manhu preparations (combined with such other foods as are permitted under the Manhu system) than would be possible on a strict anti-diabetic diet from which all carbohydrate material was excluded. The Manhu system has the merit of supplying many varieties in the diet. The ordinary monotonous dietary of diabetic patients contributes much to the discomforts of their affliction, and hardly delays the inevitable end. We therefore advise sufferers from this complaint, in so far to relax their strict observance of a non-carbohydrate diet as to try, at least for a time, the *régime* suggested by the proprietors of the Manhu foods.

### ENZYMOL.

(Fairchild Brothers and Foster, New York, U.S.A. British Agent: Burroughs, Wellcome and Co., Snow Hill, London, E.C.)

Enzymol is a solution of a proteolytic enzyme specially prepared for external application. The therapeutic properties of this solution depend on the power possessed by proteolytic ferments for digesting and rendering soluble solid proteid matter which is no longer in a living condition. In the same way that the stomach is incapable of digesting itself, so is enzymol unable to liquefy living matter, although it possesses in a high degree the power of attacking and liquefying morbid and dead tissues. Thus for instance it will rapidly liquefy and remove gangrenous tissues, unhealthy sloughs, and false membranes, such as those due to the activity of the diphtheria bacilli. The principle involved in the use of this therapeutic agent is one of the greatest value, and deserves the attention of the medical profession. Any means which can check bacterial invasion is of utmost importance in surgery. As long, however, as there exist dead tissues for bacteria to live upon, there is danger of these organisms finding their way to the source of supply. It is therefore a rational and proper treatment to remove at once all necrotic tissue by drainage and by means which liquefy solid material, for which purpose enzymol will be found invaluable.

